# The Union of Wounds

**Published:** 1898-12-17

**Authors:** 


### THE UNION OF WOUNDS.

Mr. Pickering Pick has done good service to surgery in insisting, as he has done in the Bradshaw Lecture, on the unity of all the various processes by aid of which the union of wounds takes place, and in showing that in all cases what makes wounds unite is inflammation. Obviously the whole question hinges on the definition of inflammation. Briefly and succinctly inflammation may be defined as the "response of living tissue to injury." Given in Mr. Pickering Pick's own words it is "a modification of the normal physiological processes in the various tissues of the body, resulting from the application of some irritant to the part, this process being attended or followed by the formation of a new material of a less highly organised nature than the original tissue in which the inflammation has taken place." The amount and degree of inflammation, then, must depend to large extent upon the degree, and especially on the duration, of the irritation to which the part is exposed, and herein lies the great difference between the mode of union in a septic and an aseptic wound. In the aseptic wound, however severe the injury may be, it is of momentary duration; while in the septic wound the momentary irritation of the injury is followed by a prolonged irritation arising from the growth of septic organisms. Thus the amount and degree of the resulting inflammation, and the character of the resulting tissue differ, but in each case it is inflammation which heals the wound. Of the five classical ways in which wounds are said to unite, Mr. Pick entirely discards the first, namely, that by immediate union, primary adhesion, or direct growing together of the severed surfaces, maintaining that there is and must be some connecting material to cause the cut surfaces to stick together, and, in fact, that the difference between this mode and first intention is only one of degree. He then traces out the importance of the part played by inflammation in all the other modes of union, holding that there are but two ways in which wounds unite, namely, without suppuration and with suppuration, and that the difference between these two conditions is very slight, being one of degree rather than of kind, and that they are both the result of the same morbid process going on in the part—that is inflammation. This simplifies the matter vastly.

